# Genome-Wide Association and Prediction of Male and Female Floral Hybrid Potential Traits in Elite Spring Bread Wheat Genotypes

**DOI:** 10.3390/plants10050895

**Published:** 2021-04-29

**Authors:** Samira El Hanafi, Souad Cherkaoui, Zakaria Kehel, Ayed Al-Abdallat, Wuletaw Tadesse

**Affiliations:** 1 International Center for Agricultural Research in the Dry Areas, B.P. 6299, Rue Hafiane Cherkaoui, Rabat-Institutes, Rabat 10100, Morocco; Z.Kehel@cgiar.org (Z.K.); W.Tadesse@cgiar.org (W.T.); 2 Bio-Bio Center, Physiology Plant Biotechnology Unit, Faculty of Sciences, Mohammed V University in Rabat, 4 Avenue Ibn Battouta, B.P. 1014, Rabat 10100, Morocco; s-cherkaoui@um5r.ac.ma; 3 Department of Horticulture and Crop Science, School of Agriculture, The University of Jordan, Amman 11942, Jordan; a.alabdallat@ju.edu.jo

**Keywords:** floral traits, hybrid, genomic selection, GWAS, wheat

## Abstract

Hybrid wheat breeding is one of the most promising technologies for further sustainable yield increases. However, the cleistogamous nature of wheat displays a major bottleneck for a successful hybrid breeding program. Thus, an optimized breeding strategy by developing appropriate parental lines with favorable floral trait combinations is the best way to enhance the outcrossing ability. This study, therefore, aimed to dissect the genetic basis of various floral traits using genome-wide association study (GWAS) and to assess the potential of genome-wide prediction (GP) for anther extrusion (AE), visual anther extrusion (VAE), pollen mass (PM), pollen shedding (PSH), pollen viability (PV), anther length (AL), openness of the flower (OPF), duration of floret opening (DFO) and stigma length. To this end, we employed 196 ICARDA spring bread wheat lines evaluated for three years and genotyped with 10,477 polymorphic SNP. In total, 70 significant markers were identified associated to the various assessed traits at FDR ≤ 0.05 contributing a minor to large proportion of the phenotypic variance (8–26.9%), affecting the traits either positively or negatively. GWAS revealed multi-marker-based associations among AE, VAE, PM, OPF and DFO, most likely linked markers, suggesting a potential genomic region controlling the genetic association of these complex traits. Of these markers, *Kukri_rep_c103359_233* and *wsnp_Ex_rep_c107911_91350930* deserve particular attention. The consistently significant markers with large effect could be useful for marker-assisted selection. Genomic selection revealed medium to high prediction accuracy ranging between 52% and 92% for the assessed traits with the least and maximum value observed for stigma length and visual anther extrusion, respectively. This indicates the feasibility to implement genomic selection to predict the performance of hybrid floral traits with high reliability.

## 1. Introduction

Bread wheat (*Triticum aestivum* L.) is the second most important cereal cultivated crop worldwide with 766 million tons produced in 2019 [1]. However, global climate change intensifies the effect of biotic and abiotic stresses on wheat sustainability and production. Increased wheat productivity is the ultimate objective of the breeder to meet escalating future demands. Therefore, new strategies and innovative breeding technologies might be adopted to substantially contribute to the required increase in wheat production. 

Hybrid wheat is a promising technology to increase yields worldwide. However, less than 1% of the global wheat area is planted with hybrids, mainly due to the lack of cost-effective hybrid seed process and low heterosis [2], although significant progress has been made to improve a competitive hybrid wheat breeding program. The exploitation of high-level heterosis through different hybrid systems appears more promising than ever. Easterly et al. [3] reported mid-parent heterosis for grain yield of up to 24% for hybrids developed using chemical hybridization agent (CHA), demonstrating that F1 hybrid cultivars are able to outperform inbred varieties and ensure greater economic yield. However, without baseline studies to identify parental combinations for an efficient cross-pollination system, hybrid wheat will not be attractive commercially. Moreover, to guarantee reasonable F1 hybrid seed yield, the cleistogamous nature of wheat has to be addressed. For this reason, optimizing the appropriate parents with favorable trait combinations is essential for high outcrossing ability irrespective of the employed hybridization system. Conversely, male genotypes with high level of anther extrusion for appropriate pollen release during anthesis should serve as potential male donors for receptive females with open florets for extended time periods [4]. Moreover, plant height showed its contribution in improving wheat cross-pollination ability [5,6]. It was reported previously that *Rht-B1b* and *Rht-D1b* dwarfing alleles lead to low anther extrusion that subsequently affect male floral traits [7,8]. Therefore, given these facts, breeding hybrids in such manner optimizes the exploitation of heterosis and hybrid performance. However, the intensive work and time requirements of selecting the important male and female trait combinations make the identification of hybrid parental lines more challenging. Hence, phenotypic selection might be assisted by employing molecular markers tightly linked with major QTLs for the trait of interest that could greatly accelerate and simplify the selection of wheat floral traits. However, in cases where the trait is complex and mainly governed by many loci with small effects, genomic selection or (GS) genome-wide prediction (GP) can be an alternative approach to predict the genomic estimated breeding values (GEBVs) for all the genotypes of breeding population [9]. These GEBVs allow breeders to select superior genotypes that would be suitable either as parent or for next generation advancement of the breeding program. To perform GS, a population that combines a phenotypic and genotypic data, referred to as the training population (TP), is used to train a model that takes genotypic information from a candidate population of untested individuals. This second set of individuals is considered as a validation set to estimate the prediction accuracy of the model used. GS has been extensively used in animal and plant breeding to predict traits using the molecular markers and pedigree information [9,10]. To date, little is known of the genetic architecture of floral traits or the potential use of genomics-assisted breeding that holds promise for enhancing selection for the quantitative traits. Few studies have reported the prediction accuracy of some male floral traits (anther extrusion and pollen mass) using different models [5,11,12]. In parallel, some other studies deployed genome-wide association studies (GWAS) to detect target loci associated with anther extrusion, pollen mass and anther length [5,13,14,15,16]. While the genetic architecture of male floral traits has been fairly well studied, female traits are far less understood. Our aim was therefore to identify the genetic basis of various male and female hybrid potential traits using 196 diverse wheat genotypes by: (1) performing a genome-wide association study to identify loci associated with hybrid male and female potential traits; (2) evaluating the effect of *Rht-B1* and *Rht-D1* on the floral traits; and (3) investigating the potential of genome-wide prediction (GP) for the evaluated floral traits. 

## 2. Results

We observed a wide phenotypic variation for the assessed floral traits among the 196 diverse bread wheat genotypes (Table S1). The distribution of the evaluated traits showed normal distributions, and the estimated variabilities, correlations and heritabilities were reported previously by El Hanafi et al. [4] (Table S1). We showed that absolute values of phenotypic correlations among the evaluated floral traits ranged between 0.3 and 0.90 (*p* < 0.001) with the highest correlation recorded between anther extrusion and pollen mass (Table S2) [4]. The resulting BLUEs were used to run GWAS and genomic prediction.

### 2.1. Population Structure and Linkage Disequilibrium

Population structure (Q) was investigated by Bayesian-based structure analysis using 102 non-redundant markers across the whole genomes. The population structure of the 196 genotypes showed three subpopulations. A similar clustering result was obtained using discriminant analysis of principal components (DAPC) and principal component analysis (PCA). A detailed analysis was described by Tadesse et al. [17].

In total, 10,477 polymorphic SNP markers out of 12,725, retained after removing SNPs with >10% missing data and MAF < 5%, were used to determine LD between all pairs of markers. The LDs for locus pairs and the scatter plot of LD (R^2^) as a function of the intermarker distance (Mbp) for all genotypes were described by El Hanafi et al. [18] and Tadesse et al. [17]. The plot of the LD estimates (R^2^) as a function of the intermarker genetic distance (Mbp) within the same chromosome for all genotypes indicated a clear LD decay with genetic distance. The baseline intersected with the spline curve at 6 Mbp based in the average R^2^ = 0.23, as shown by El Hanafi et al. [18]. 

### 2.2. Marker Trait Associations

**Figure 1:**
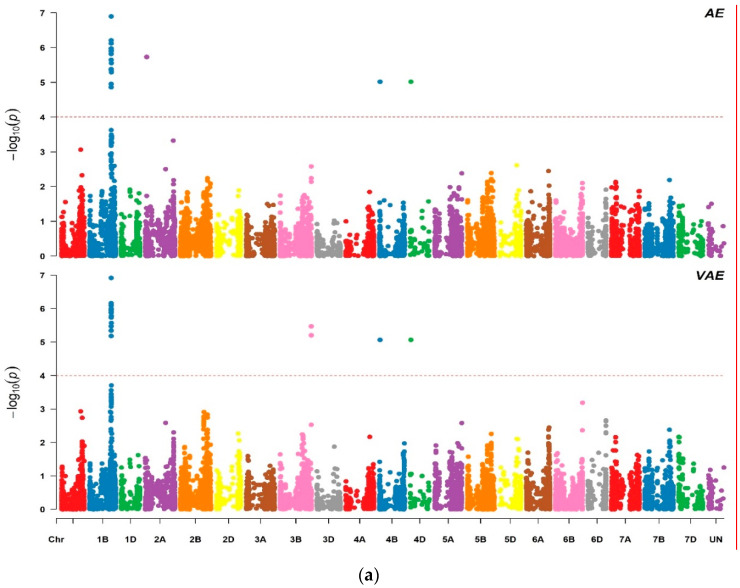

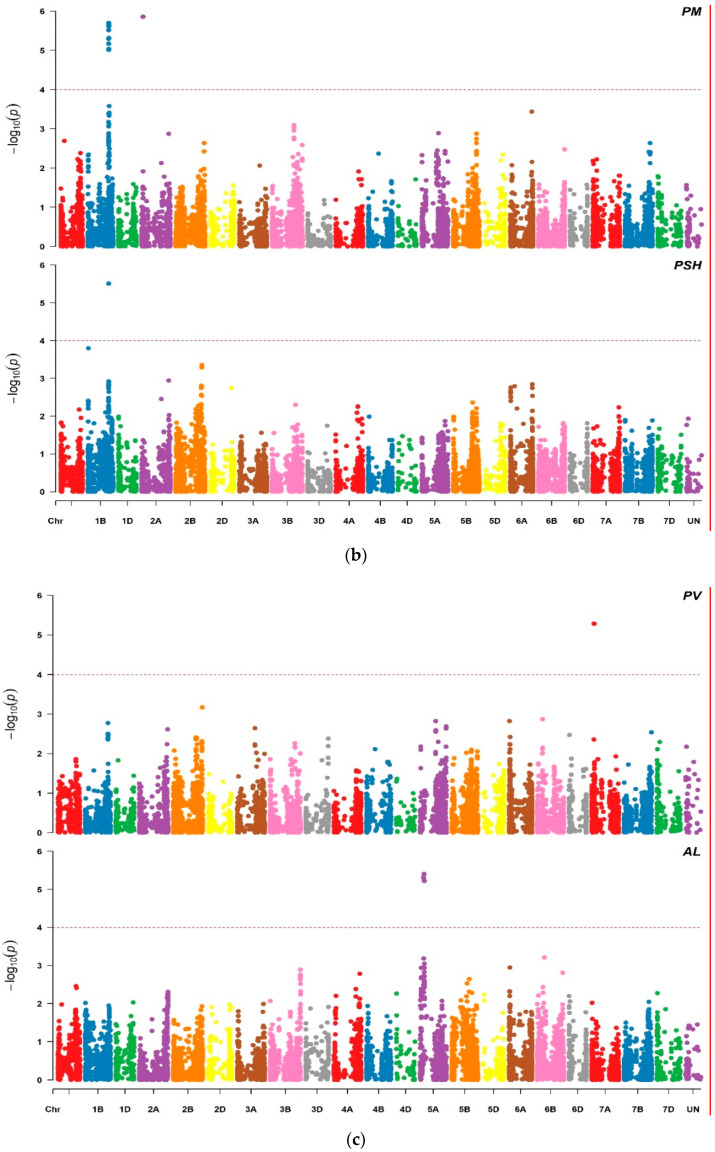

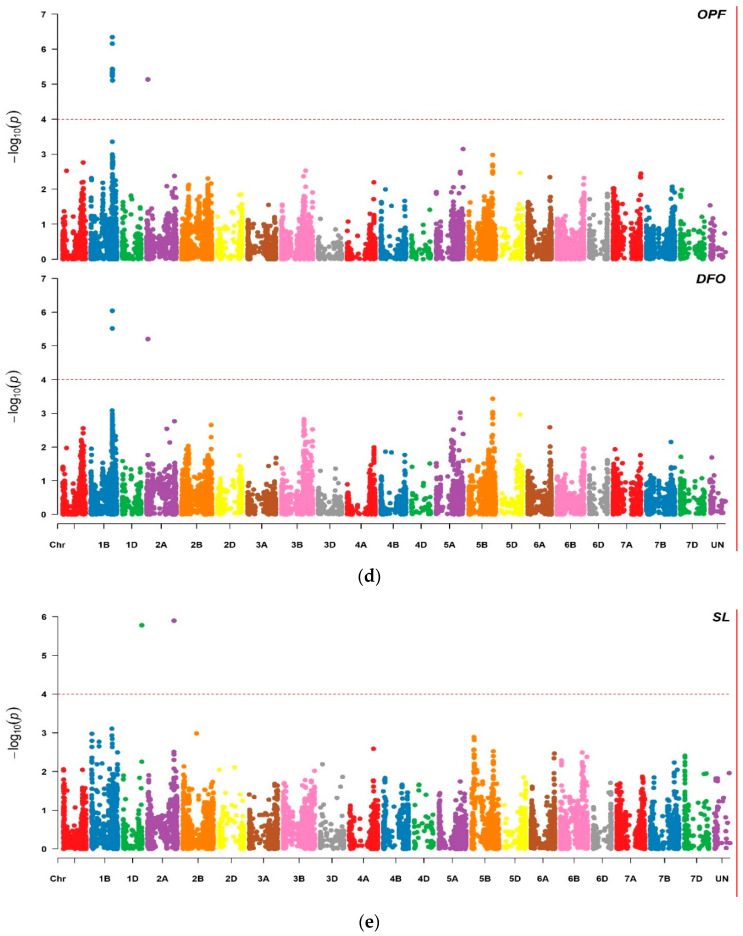
Manhattan plots of genome-wide association analysis for the evaluated floral traits: (**a**) AE and VAE; (**b**) PM and PSH; (**c**) PV and AL; (**d**) OPF and DFO; and (**e**) SL. *p* values are shown on a −log10 scale and the significant markers are above the threshold FDR ≤ 0.05. Trait abbreviations are given in Table 1 and Table 2.

Extensive SNP genotyping was used to analyze the 196 ICARDA spring bread wheat genotypes. Out of the 12,725 markers obtained, 10,477 SNP markers were used to identify markers associated to the floral traits using Q + K MLM method. Genome-wide association mapping revealed 70 significant markers at FDR ≤ 0.05. For the male floral traits, 17 markers were associated with anther extrusion (AE), 18 with visual anther extrusion (VAE), 14 with pollen mass (PM), 3 with anther length (AL), 1 with pollen shedding (PSH) and 1 with pollen viability (PV) (Table 1 and Table 2 and Figure 1). For the female potential traits, 11, 3 and 2 MTAs were detected for openness of the floret (OPF), duration of floret opening (DFO) and stigma length (SL), respectively (Table 1 and Table 2 and Figure 1). The percentage of the phenotypic variance ranged from 8% to 26.93%. Marker *wsnp_Ex_c23235_32471767* on chromosome 1B explained the highest phenotypic variation of R^2^ = 26.93% for anther extrusion. Most of the significant markers showed an association with at least three different traits (Table 1). *Kukri_rep_c103359_233* and *wsnp_Ex_rep_c107911_91350930* showed associations with AE, VAE, PM, OPF and DFO. Seven markers (*wsnp_Ex_c13109_20725640*, *wsnp_Ex_c23235_32471767*, *wsnp_Ex_rep_c107911_91350866*, *wsnp_Ex_c21198_30327016*, *wsnp_Ku_rep_c89089_82009060*, *IAAV3802* and *Ex_c11930_509*) were significantly associated with AE, VAE, PM and OPF. MTA analysis based on semi-dwarfing genes *Rht-1* (*Rht-B1* and *Rht-D1*) detected an association of *Rht-D1b* with both AE and VAE, which explained 20.57% and 17.34% of the phenotypic variation, respectively. There was no significant association with *Rht-B1*. A small negative effect was observed for the dwarfing allele *Rht-D1b* on AE and VAE of −3.12 and −0.59, respectively (Figure 1 and Table 1 and Table 2).

We investigated the relationship between the number of favorable alleles and the performance of the genotypes. Of the 17 significant markers associated with AE, 11 have a favorable effect (an increasing effect on AE) while 6 have an unfavorable effect (decreasing effect on AE). Sixty-six genotypes harbored the maximum favorable allele and five the maximum unfavorable one (Table S3a). The best performing genotype carrying the maximum number of favorable alleles was SOMAMA-9/ICARDA-SRRL-2 with the high AE value of 90.03%. We investigated the relevance of the addition of every allele in predicting AE performance by correlating the cumulative number of favorable and unfavorable alleles on the AE-BLUEs values. There was significant correlation between the AE-BLUEs values and their respective favorable and unfavorable alleles with R^2^ = 0.16 and R^2^ = 0.18 (*p* < 0.001), respectively. 

Alleles per marker for VAE varied between 24 and 139. *Rht-D1b* was the marker with the highest number of observed alleles. SERI.1B//KAUZ/HEVO/3/AMAD/4/ATTILA//PSN/BOW/3/ATTILA/5/KAUZ’S’/SHUHA-15 and SOMAMA-9/ICARDA-SRRL-2, with the best VAE performances of 9.45 and 9.3, respectively, were the genotypes having the maximum favorable alleles (12). The linear regressions of the number of favorable and unfavorable alleles per genotypes versus the VAE-BLUEs values showed significant association with R^2^ = 1.6 and R^2^ = 1.5 (*p* < 0.001), respectively (Table S3b).

GWAS identified 14 significant markers associated with PM at FDR < 0.05, which were all commonly present with those associated with AE (Table 1). Ten markers had favorable additive effects, while five were unfavorable (Table S3c). The highest number of observations per marker was associated with those having unfavorable effect. The significant markers explained phenotypic variances (R^2^) ranging from 21.29% to 25%. Again, SOMAMA-9/ICARDA-SRRL-2 appears among the best genotypes having the highest number of favorable alleles (six) and showing good pollen mass performance (41.11 mg). The linear regressions of the number of favorable and unfavorable alleles per genotypes versus the PM-BLUEs values showed significant associations with R^2^ = 1.7 and R^2^ = 1.6 (*p* < 0.001), respectively.

GWAS identified 11 significant markers associated with OPF on chromosomes 1B (10) and 2A (1), of which 12, 11 and 12 were common with AE, VAE and PM, respectively (Table 1). The significant markers explained phenotypic variances (R^2^) ranging from 23.48% to 24.53%. Eighty-six genotypes had the maximum cumulative number of favorable allele effect (six) (Table S3d). SOMAMA-9/ICARDA-SRRL-2 is the best genotype in terms of high OPF phenotypic values and carrying the highest number of favorable alleles. Linear regression on the relationship between the OPF-BLUEs values and the number favorable and unfavorable alleles showed significant associations with R^2^ = 1.13 and R^2^ = 1.19 (*p* < 0.001), respectively.

Besides the female floral traits, GWAS detected three significant markers associated to duration of floret opening (DFO) with phenotypic variance of 18.7% each located in 1B (two) and 2A (one) (Table 1). The same markers were present in association with AE, PM and OPF. SOMAMA-9/ICARDA-SRRL-2 and HAAMA-17/ANGI-2 are among the genotypes having the best OPF performance and carrying the highest number of favorable alleles (Table S3e). 

### 2.3. Genome-Wide Prediction of the Potential Floral Traits

We evaluated genome-wide prediction of the potential floral traits with relevance for hybrid breeding in wheat. The accuracies of genome-wide prediction were estimated by using tenfold cross-validation based on the same set of phenotypic and genomic data used for association mapping analysis. Low to moderate genomic heritability (h^2^) was observed for the evaluated floral traits with the highest value (0.30) recorded for anther length while stigma length showed the lowest heritability (0.08) (Table 3). The prediction ability ranged between 0.45 for anther extrusion and 0.14 for stigma length across years. The prediction accuracy of the model for hybrid wheat floral traits were high for all evaluated floral traits, ranging between 0.92 for visual anther extrusion and 0.52 for stigma length. 

## 3. Discussion

Hybrid wheat breeding technology has great potential to raise wheat production, particularly in the context of global climate change and future food security. The maximum heterosis achieved can assess the usefulness and efficiency of a hybrid wheat program. However, the stringent autogamous nature of wheat presents a bottleneck for suitable seed production system. The ability of enhanced outcrossing might be achieved by choosing specific parental lines for best hybrid combination. Selection for potential hybrid wheat traits such as anther extrusion, pollen mass, degree and duration of floret opening is challenging and time consuming. Molecular tools are widely considered to hold promise for the dissection of genomic regions harboring genes/markers for hybrid traits. In this study, we evaluated the potential of genomic approaches in identifying genes/QTL that could be effectively used to fine-tune the floral traits and greatly simply redesigning wheat flower.

### 3.1. Marker Traits Associations

While hybrid wheat programs have operated for several decades and phenotyping floral characteristics traits have been fairly studied, little is known of the genetic mechanisms controlling male and female flower development. Marker–trait associations (MTAs) were identified for the potential floral hybrid wheat traits by using a mixed linear model considering both kinship (K) and population structure (Q) matrices as covariates. In total, 70 markers were associated with various evaluated traits. For the floral associated markers, 17 and 18 MTAs were linked to AE and VAE, respectively, of which 14 markers were in common. The phenotypic variance explained by these markers ranged 17.33–26.92%, which is greater than what was reported by Muqaddasi et al. [16] and Boeven et al. [5]. The MTAs associated with AE and VEX located on chromosome 1B are in tight LD with each other and located at close distances (566–571 Mbp), making this genomic region very important and warranting further investigation. The consistently significant markers carrying the maximum favorable alleles confer high performance for that specific trait, indicating the importance of accumulating favorable alleles to improve the potential floral traits key success for hybrid wheat production.

The comparison of marker intervals with previously reported studies is challenging because most used different marker systems (AFLP, DArT and SSR markers) and genetic linkage maps (RIL, DH and F2 populations) [7,19,20,21]. Few recent association mapping studies have been published using DArT and GBS markers [13,16,22] or SNP markers [5,11,12]. The marker *wsnp_Ex_c5101_9053178* on chromosome 1B found associated with AE and VAE was reported previously by Boeven et al. [5]. Similarly, the two markers *Excalibur_c20309_539* and *Kukri_rep_c110544_248* (3B) associated with VAE were previously reported by Muqaddasi et al. [15]. Other than those, all markers identified were different despite the high heritability of the trait; this might be explained by the strong dependence of anther extrusion on the genetic background and/or other related floral traits such as flower gapping and spikelet spacing.

The complex nature of anther extrusion indicates the trait is governed by many loci with small to large effects and explains the considerable level of phenotypic variance. A similar conclusion has been drawn in many studies [5,7,16,19,20,23].

**Figure 2:**
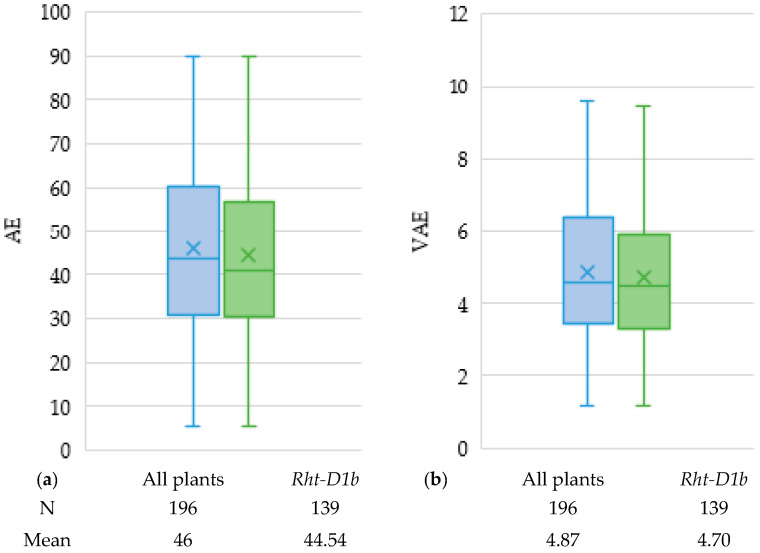
Box plots showing AE (**a**) and VAE (**b**) for genotypes carrying dwarfing gene allele *Rht-D1b*. N represents the total number of genotypes for the whole set (bleu) and the genotypes carrying *Rht-D1b* (green) and the mean values associated with each group. AE, anther extrusion; VAE, visual anther extrusion.

Semi-dwarfing allele *Rht-D1b* showed an association with both AE and VAE, which explained, respectively, 20.57% and 17.33% of the phenotypic variance. The candidate gene *Rht-B1* was not significantly associated with any of the evaluated floral traits, probably due to the very low number of lines carrying the mutant type of *Rht-B1,* or it might also be attributed to the different mechanism of action of the semi-dwarfing gene under the influence of the surrounding genetic backgrounds [5,23]. On average, lines carrying the dwarfing allele *Rht-D1b* had a reduced AE by 1.45% and showed reduced VAE by almost two scoring points (Figure 2). In line with previous studies [24,25], our results thus provide strong evidence for the negative effect of *Rht-D1b* on anther extrusion. However, it is important to note that the observed effect might be attributed not only to the allelic variation of the gene itself but also to significant epistatic interaction with other closely linked genes in the locus [26,27]. Some other GWAS studies did not reveal an association of AEX or VAEX with *Rht-B1* alleles [5,11,12,15,16]. Parallelly, He et al. [8] did not show the effect of *Rht-D1* on anther extrusion in a population of 131 RILs. Nevertheless, high AE wheat accessions carrying semi-dwarfing alleles of *Rht-B1b* or *Rht-D1b* do exist [28].

It has been demonstrated that dwarfing genes affect not only the height but also some other floral associated traits. We showed weak correlation between plant height and anther length using the same panel in El Hanafi et al. [4], but we did not observe any association between *Rht* genes and anther length. In the same context, Okada et al. [29] found that *Rht-1* had significant association with anther length, anther extrusion, plant height and spike length. Moreover, Cheng et al. [30] reported that *Rht-D1* encodes DELLA proteins that act as repressors of plant growth in the presence of gibberellin acid (GA). Semi dwarfing alleles have gibberellin-insensitivity resulting in reduced plant height and low anther extrusion at the same time. This shows that *Rht* genes are involved in multiple floral traits and, therefore, can be used as candidate genes for improved anther extrusion. However, it would be interesting to test additional plant height genes for better understanding of their effect on anther extrusion and other floral traits, and it might subsequently offer choices to take full advantage of the existing dwarf genetic resources and to use them in designing superior hybrids in breeding programs. 

More recently, anther extrusion has been the focus of several linkage mapping for fusarium head blight (FHB) resistance since high extruded anthers showed tight association with lower FHB infection [7,8,20,22,31,32]. Several studies found that the peaks of FHB QTL were constantly located at the *Rht-D1* locus on 4DS, which is in concordance with our findings. Similarly, the two markers *Excalibur_c20309_539* and *Kukri_rep_c110544_248* on chromosome 3B at very close distances were associated with VAE and located in the *QFhs.ndsu-3B* region, which is a major QTL for FHB resistance [33]. Considering the close relationship of VAE and FHB resistance, these markers could be proposed as promising morphological markers for FHB resistance and vice versa.

Pollen mass, another important male floral trait, was shown to be taken as proxy for an initial selection of superior genotypes in early generations [5]. However, considering its intensive work and time requirements, we therefore focused in this study on understanding its genetic basis in order to identify associated markers and thereby pave the way for a systematic exploitation of heterosis in wheat. Our genome-wide association mapping revealed 14 MTAs associated with pollen mass with a small effect observed and accounting for maximum 23.76% of the phenotypic variance. Among these markers, 14 and 13 markers were implicated as sites for AE and VAE, respectively. A recent association mapping study for PM in winter bread wheat detected six QTL for pollen mass, among which *Rht-D1* was the most strongly associated locus [5]. Conversely, our study did not show similarities between the markers previously detected or an association of PM with *Rht* alleles. Therefore, the absence of a major marker and small effect markers suggests the complex genetic architecture of pollen mass.

Pollen shedding, taken as a proxy, appears to be a promising trait, allowing indirect selection of the best performing male and female associated traits [4]. Being an important determinant for outcrossing, profuse pollen shedding outside the florets is important when there is a high rate of extruded anthers. This has been demonstrated by the high correlation between released pollen mass and pollen shedding [4,23]. However, GWAS did reveal one marker *Excalibur_c10496_651* on 1B associated to PSH commonly identified for PM, AE and VAE. The small number of markers with no major gene associated to PSH more likely suggests a quantitative inheritance and thus a complex genetic architecture underlying the trait pollen shedding. 

The importance of pollen viability as a factor in enhancing the out-crossing ability in wheat has long been recognized. However, to our knowledge, assessment of the viability of wheat pollen has been done using staining methods, in vitro germination or impedance flow cytometry [34]. However, except these studies, the genetic basis of pollen viability has not been investigated in great detail up to now. Therefore, we performed GWAS, aiming to find tightly linked markers to pollen viability. *Excalibur_rep_c109881_701* (7A) was the only marker showing an association with pollen viability and contributing 19.35% of the observed phenotypic variation. The small number of associated markers might be in part due to gaps in the genome with poor marker coverage or the complex genetic architecture underlying this trait.

Adequate cross pollination attributes are essential for hybrid development. Many reports have emphasized the importance of wheat anthers and stigma size as favorable pollinator traits in grain setting, a crucial factor determining the success of hybrid wheat programs [23,35,36]. Work focusing on the genetic control of anther and stigma length in wheat is very limited. Therefore, identification of genomic regions associated with AL through genome-wide association study would help in this regard. In the current study, MLM analysis revealed three markers associated with AL on 5A and two other markers associated with stigma length on 1D and 2A. To date, few genes have been identified associated effectively in increasing wheat AL, while the genetic basis of stigma length is still unknown. Hence, it is currently difficult to align and compare the identified markers. Cheng et al. [14] revealed seven major markers that were associated with AL which did not overlap with any of the identified markers. It has also been observed that the addition of rye chromosome 4R to wheat increases anther length by 16% [37]. This finding can greatly enhance pollination in wheat. Besides, further studies are required to dissect the novel genes related to anther and stigma length, which can increase our understanding of the underlying genetic mechanisms of AL and SL that represent key traits for the successful production of hybrid wheat seeds.

The genetic architecture of female traits is far less dissected. Previous studies have hinted at wide variability among female hybrid traits such as the degree and the duration of floret opening. However, it is often challenging to address these traits due to the intensive work and time requirements. Therefore, we opted to detect the genomic regions harboring markers associated to openness of the florets and duration of floret opening to substantially select the superior female parents. GWAS revealed 11 and 3 MTAs associated with OPF and DFO, respectively. Among these markers, *wsnp_Ex_rep_c103167_88182254* (2A) was reported to be associated with OPF DFO, PM and AE. *Kukri_rep_c103359_233* and *wsnp_Ex_rep_c107911_91350930* on chromosome 1B were potentially linked to OPF, DFO, PM, AE and VAE, suggesting a potential genetic association that controls these complex traits.

Moreover, it has been demonstrated that wheat plants with narrow flower opening (cleistogamous) and/or short duration of floret opening will have a lower incidence of FHB by reducing the area and period in which Fusarium spores can enter the floret and initiate infection [32,38,39]. Therefore, the genomic regions of the markers associated to OPF and DFO merit further research and thus the validation of the markers associated to FHB is particularly valuable and may allow indirect selection for these two female traits or vice versa.

### 3.2. Genome-Wide Prediction of Hybrid Floral Traits

The potential of genomic selection for hybrid floral traits have previously only been conducted for anther extrusion and pollen mass [5,11,12]. Boeven et al. [5] evaluated different schemes to perform genomic selection using 209 winter wheat lines and reported prediction abilities of 0.3, 0.6 and 0.4 for VAE, counted AE and PM, respectively, using rrBLUP model. Inclusion of weighted effect *Rht* loci via based on weighted ridged regression BLUP (wrrBLUP) increased the prediction abilities to 0.5, 0.7 and 0.57 for VAE, counted AE and PM, respectively. In [11], prediction accuracy was 0.62 for VEX using rrBLUP medel. Recently, Adhikari et al. [12] performed genomic prediction for AE using 603 advanced parental lines from the CIMMYT hybrid breeding program applying seven models of increasing complexity and using three cross-validation scenarios. High prediction accuracy was observed for AE and VEX with 0.45 and 0.73, respectively, where all lines in one environment were fully predicted by the other environments. However, prediction accuracy of the main effect model comparable to the previous studies was in the range of 0.42–0.44 across environments for counted AE and 0.33–0.46 for VEX. In the current study, using tenfold cross-validation, high prediction accuracy was recorded for AE, VAE and PM with 0.85, 0.92 and 0.83, respectively, demonstrating the feasibility of using genomic selection for quick identification of potential hybrid parents. Pollen shedding, anther length, openness of the floret and duration of floret opening have also shown high prediction accuracy with 0.86, 0.73, 0.85 and 0.76, respectively, indicating that these traits can be predicted with high reliability. Stigma length showed on the one hand moderate prediction accuracy of 0.52, indicating that genomic selection can be useful to preselect promising female lines with acceptable accuracy, and on the other hand very low genetic heritability. However, subsequent field/lab test might be needed to verify the suitability of the preselected lines for hybrid wheat production. The low to moderate predictive ability and genomic heritability recorded in the current study might be due to the small population size, the genetic architecture of the trait of interest and/or the impact of adding fixed effects (genes/QTL) [40].

## 4. Materials and Methods

### 4.1. Plant Materials and Phenotyping

This study was based on a diverse set of 196 elite genotypes from ICARDA bread wheat breeding program (Table S4). The panel and the trial protocol were previously described in detail by El Hanafi et al. [4]. The genotypes were first evaluated under plastic house conditions at ICARDA Rabat Guich, Morocco (33°97′ N, 6°86′ W, 57 masl) in 2016 and then were grown in 3 m^2^ plots in an augmented design for two more years during the 2017 and 2018 cropping seasons at Marchouch Station, Morocco (33°36′ N, 6°43′ W, 394 masl).

During the growth period, various floral traits were assessed in all environments, as described by El Hanafi et al. [4]: (1) Visual anther extrusion (VAE) was recorded on a scale from 1 to 9 (1 = no anthers extruded, 9 = maximum anther extrusion). (2) Anther extrusion (AE) was based on the count of the remained anthers inside the florets of the spikes harvested 5–7 days post flowering peak. (3) Pollen mass (PM) was recorded to estimate the released pollen from 10 randomly selected spikes. (4) Pollen shedding (PSH) was visually scored on a scale from 1 to scale (1 = no pollen shedding and 9 = maximum pollen shedding). (5) Pollen viability (PV) was determined by the I-KI method based on a count of 100 pollen grains [41]. (6) Openness of the floret (OPF) was recorded as separation angle between the glumes of first two florets of a spikelet. (7) Duration of floret opening (DFO) was visually recorded in minutes from the time of opening to closing of florets by a digital video. (8) Anther length (AL) was scored at flowering stage as the average of three anthers belonging to a lateral floret of the three central spikelets of a spike using image analysis software ImageJ [26]. (9) Stigma length (SL) was determined using as average length of two stigmas of one lateral floret from three central spikelets of a spike using image analysis software ImageJ [26].

### 4.2. Genotyping by SNP Markers

Genomic DNA from two-week-old bulked leaf samples were frozen in liquid nitrogen and stored at −80 °C before DNA extraction. The DNA was carried out according to Ogbonnaya et al. [27], after which 10 μL of a 100 ng μL^−1^ DNA of each sample were sent to Trait Genetics, Germany [42], as a commercial service provider for whole genome scan using Single nucleotide polymorphism (SNP) markers. In total, 15,000 SNP markers were used to genotype the 196 wheat genotypes; 10,477 SNP markers resulted after discarding SNPs with minor allele frequency of <5% and missing values cut off (10%).

### 4.3. Population Structure and Linkage Disequilibrium

The genetic structure of the 196 genotypes was assessed with 102 unlinked SNP markers distributed across the wheat genome with at least two loci on each wheat chromosome using the STRUCTURE software [43]. A detailed analysis of the population structure and discriminant analysis of principal components (DAPC) can be found in the work of Tadesse et al. [17]. 

In total, 10,477 out of 12,725 SNPs, retained after removal of those with MAF < 5% and missing values over 10%, were used to study linkage disequilibrium (LD). TASSEL 5.0 [44] was used to estimate LD as squared allele frequency correlation estimates (R^2^) and measure the significance of R^2^ at *p*-values for each pair of loci on different chromosomes (interchromosomal LD) and within the same chromosome (intrachromosomal LD).

### 4.4. Association Mapping

Genome-wide association studies (GWAS) were utilized to find target loci associated with higher anther extrusion, pollen mass, maximum pollen shed outside the anthers, long and viable pollen grains, wide florets opening and hairy stigmas receptive for extended periods.

Marker trait association analysis was performed using mixed linear model (MLM) with TASSEL 5.0 software [44], applying both kinship matrix (K) and population structure (Q) matrices as covariates. Individuals with >10% missing SNP calls and markers with >10% missing and <5% minor allele frequency (MAF) were removed. To eliminate linear dependency between columns of the Q matrix, the last column was removed prior to GWAS. To check whether the model we implemented was sufficiently stringent to control the population stratification and spurious associations, quantile–quantile (qq) plots were drawn to compare the distribution of observed versus expected *p*-values of all the SNPs at −log10 scale [45]. 

For the detection of significant marker trait associations (MTAs), we applied a significance threshold of false discovery rate (FDR) ≤ 0.05. The −log10 (*p*) values were displayed in the form of Manhattan plots using R library “CMplot” package in R 3.3.1 [46].

### 4.5. Genomic Prediction

The genomic prediction (GP) was performed based on a two-stage analysis with weights. A genomic relationship matrix G was generated using 10,478 markers resulting from filtering and following the methodology described by Endelman et al. [47] using rrBLUP [48] package in R [46]. Genomic heritability (h^2^) was computed as the ratio between the genetic variance due to markers over the sum of the genetic variance plus the error variance. This was done using the complete dataset to estimate variance components (additive and residuals) and hence the genomic heritability. Genomic best linear unbiased prediction (G-BLUP) was used to perform genome wide predictions. Models were performed using ASReml-R version 3.0 [49].

The evaluation of the different GP models for each trait was done by calculating the predicted ability (PA) and the prediction accuracy (Pacc) of the genomic estimated breeding values (GEBV) by using 10-fold cross-validation with 20% of the genotypes as validation set and the remaining 80% as training set for the models. PA was estimated as the correlation between the adjusted phenotypic value and the GEBV from the GP model. In addition, Pacc of GEBV is the correlation between the true genomic breeding value and the predicted breeding value, which was estimated according to Equation (1):

(1) $$\mathrm{Pacc}=\frac{\mathrm{PA}}{\sqrt{h^{2}}}$$

## 5. Conclusions

Considering the high heritabilities coupled with high genetic advance, conventional methods based on direct or visual selection of phenotypes have contributed significantly to improving floral traits with the relevance for hybrid breeding. However, the assessment of these traits is challenging and time-consuming. To cope with this constraint, we employed genome-wide association study to reveal the genetic basis of various male and female hybrid potential traits. Many markers with small to large effects were detected, indicating the genetic complexity of the evaluated traits. Within this context, the significant markers with medium to large effects can be applied in hybrid breeding via marker-assisted selection. However, in the case of minor genes/QTL associated to the trait of interest, phenotypic selection remains most cost-effective and promises a high selection response. Nevertheless, these minor effect genes might be included with the aim of improving the related trait. In parallel, genome-wide prediction appears promising to predict most of the hybrid floral traits with high accuracy for the highly quantitative traits that are controlled by small- to large-effect genes/QTLs. 

## Figures and Tables

**Table 1:** Significant common markers associated with anther extrusion, visual anther extrusion, pollen mass, openness of the floret and duration of floret opening at FDR ≤ 0.05.

Marker	Chr	Position (Mbp) ^a^	AE			VAE			PM			OPF			DFO		
			−Log10 (*p*)	Effect	%R^2 b^	−Log10 (*p*)	Effect	%R^2^	−Log10 (*p*)	Effect	%R^2^	−Log10 (*p*)	Effect	%R^2^	−Log10 (*p*)	Effect	%R^2^
*Rht-D1b*	4D	-	5.02	0.18	20.58	5.07	0.05	17.34									
*wsnp_Ex_rep_c103167_88182254*	2A	33.04	5.73	−11.40	26.08				5.86	−3.03	23.76	5.13	−3.03	24.07	5.20	−10.1	18.7
*Tdurum_contig30517_578*	1B	566.84	5.37	12.88	25.34	5.18	1.22	20.95	5.01	3.54	21.98						
*Tdurum_contig30517_310*	1B	566.84	5.65	13.51	25.87	5.34	1.26	21.16				5.30	3.75	24.24			
*BS00062724_51*	1B	567.71	6.13	14.17	26.21	6.10	1.38	22.39	5.64	3.69	22.79						
*Kukri_rep_c103359_233*	1B	568.52	6.90	13.57	26.28	5.88	1.32	22.33	5.65	3.64	22.99	6.34	3.64	24.38	6.04	11.37	18.68
*wsnp_Ex_c13109_20725640*	1B	568.53	4.86	−14.10	26.53	5.96	−1.37	22.52	5.67	−3.60	23.24	6.16	−3.60	24.00			
*wsnp_Ex_c23235_32471767*	1B	568.57	6.21	14.35	26.93	6.16	1.39	22.93	5.70	3.74	23.28	5.44	3.74	24.53			
*Excalibur_c10496_651*	1B	568.97	5.38	−12.80	24.75	5.74	−1.33	21.64	5.17	−3.01	21.88						
*wsnp_Ex_c23230_32466568*	1B	568.97	4.96	12.68	25.56	5.48	1.23	21.62	5.30	3.37	22.39						
*wsnp_Ex_rep_c107911_91350866*	1B	570.28	5.97	13.73	26.52	6.92	1.33	22.44	5.66	3.63	23.03	5.31	3.63	24.24			
*wsnp_Ex_rep_c107911_91350930*	1B	570.28	5.90	13.57	26.28	5.88	1.32	22.33	5.65	3.64	22.99	5.34	3.64	24.38	5.52	11.36	18.67
*wsnp_Ex_c21198_30327016*	1B	570.29	5.98	13.79	26.47	6.00	1.35	22.66	5.52	3.61	22.98	5.27	3.61	24.24			
*wsnp_Ku_rep_c89089_82009060*	1B	570.30	5.82	−13.50	25.37	5.93	−1.34	21.90				5.23	−3.60	23.48			
*IAAV3802*	1B	571.06	5.56	−13.30	25.42	4.79	−1.30	21.73	5.31	−3.52	22.14	5.41	−3.52	23.57			
*wsnp_Ku_c13043_20902807*	1B	571.06	5.29	12.56	24.50	5.57	1.29	21.16	5.03	3.02	21.49						
*Ex_c11930_509*	1B	571.18	5.93	−3.52	24.10	5.98	−1.36	22.67	5.62	−3.52	23.22	5.11	−3.52	24.10			
*Excalibur_c20309_539*	3B	819.86				5.47	1.55	21.16									
*Kukri_rep_c110544_248*	3B	819.89				5.20	1.54	21.09									

^a^ Physical position based on the IWGSC RefSeq. 1.0; ^b^ R^2^, phenotypic variation; AE, anther extrusion; VAE, visual anther extrusion; PM, pollen mass; OPF, openness of the floret; DFO, duration floret opening.

**Table 2:** Significant markers associated with pollen shedding, stigma length, anther length and pollen viability at FDR ≤ 0.05.

Marker	Trait	Chr	Physical Position (Mbp) ^a^	−Log10 (*p*)	Effect	%R^2 b^
*Excalibur_c10496_651*	PSH	1B	568.97	5.51	−1.04	22.10
*BS00063511_51*	SL	1D	485.71	5.78	0.39	8.01
*Kukri_c62142_683*	SL	2A	692.64	5.90	0.24	9.55
*BS00064387_51*	AL	5A	87.40	5.30	0.20	17.86
*wsnp_Ra_c10053_16636851*	AL	5A	99.02	4.40	0.20	17.86
*wsnp_Ku_c328_679106*	AL	5A	104.23	5.22	0.20	17.86
*Excalibur_rep_c109881_701*	PV	7A	65.97	5.29	5.40	19.35

^a^ Physical position based on the IWGSC RefSeq. 1.0; ^b^ R^2^, phenotypic variation; PSH, pollen shedding; SL, stigma length; AL, anther length; PV, pollen viability.

**Table 3:** Genomic heritability, prediction ability and prediction accuracy of the assessed floral traits.

Trait	h^2^	PA	Pacc
VAE	0.21	0.43	0.92
PSH	0.24	0.42	0.86
AX	0.28	0.45	0.85
DFO	0.2	0.38	0.85
AX	0.28	0.45	0.85
PM	0.24	0.41	0.83
OPF	0.27	0.39	0.76
AL	0.3	0.4	0.73
SL	0.08	0.14	0.52

PA, prediction ability; Pacc, prediction accuracy; AE, anther extrusion; VAE, visual anther extrusion; PM, pollen mass; OPF, openness of the floret; DFO, duration floral opening; PSH, pollen shedding; SL, stigma length; AL, anther length; PV, pollen viability.

## Data Availability

Data is contained within the article or supplementary material.

## Supplementary Materials

The following are available online at https://www.mdpi.com/article/10.3390/plants10050895/s1, Table S1: Summary statistics for the evaluated traits. Table S2: Pearson correlation coefficient and their levels of significant between traits. Table S3: Relationship of favorable and unfavorable alleles with the trait performance. Table S4: List of the 196 elite spring bread wheat genotypes used in this study.
